# Role of Spexin in White Adipose Tissue Thermogenesis under Basal and Cold-Stimulated Conditions

**DOI:** 10.3390/ijms25031767

**Published:** 2024-02-01

**Authors:** Sabrina E. Gambaro, María G. Zubiría, Alejandra P. Giordano, Patricia F. Castro, Carolina Garraza, Alejandro E. Harnichar, Ana Alzamendi, Eduardo Spinedi, Andrés Giovambattista

**Affiliations:** 1 Neuroendocrinology Laboratory, Multidisciplinary Institute of Cellular Biology (IMBICE, CICPBA-CONICET-UNLP), La Plata 1900, Argentina; sgambaro@imbice.gov.ar (S.E.G.); gzubiria@imbice.gov.ar (M.G.Z.); giordanoalejandrap@gmail.com (A.P.G.); castro.patricia77@gmail.com (P.F.C.); caro.garraza9@gmail.com (C.G.); aeharnichar@gmail.com (A.E.H.); aalzamendi@imbice.gov.ar (A.A.); 2 Biology Department, School of Exact Sciences, La Plata National University, La Plata 1900, Argentina; 3 CENEXA (UNLP-CONICET), La Plata Medical School-UNLP, Calles 60 y 120, La Plata 1900, Argentina; spinedi@cenexa.org

**Keywords:** Spexin, thermogenesis, beige adipocytes, white adipose tissue, UCP1

## Abstract

Spexin (SPX) is a novel adipokine that plays an emerging role in metabolic diseases due to its involvement in carbohydrate homeostasis, weight loss, appetite control, and gastrointestinal movement, among others. In obese patients, SPX plasma levels are reduced. Little is known about the relationship between SPX and white adipose tissue (WAT) thermogenesis. Therefore, the aim of the present study was to evaluate the role of SPX in this process. C57BL/6J male mice were treated or not with SPX for ten days. On day 3, mice were randomly divided into two groups: one kept at room temperature and the other kept at cold temperature (4 °C). Caloric intake and body weight were recorded daily. At the end of the protocol, plasma, abdominal (epididymal), subcutaneous (inguinal), and brown AT (EAT, IAT, and BAT, respectively) depots were collected for measurements. We found that SPX treatment reduced Uncoupling protein 1 levels in WAT under both basal and cold conditions. SPX also reduced *cox8b* and *pgc1α* mRNA levels and mitochondrial DNA, principally in IAT. SPX did not modulate the number of beige precursors. SPX decreased *spx* levels in IAT depots and *galr2* in WAT depots. No differences were observed in the BAT depots. In conclusion, we showed, for the first time, that SPX treatment in vivo reduced the thermogenic process in subcutaneous and abdominal AT, being more evident under cold stimulation.

## 1. Introduction

Spexin (SPX), a novel adipokine, is a peptide of 14 amino acids that has been discovered using bioinformatic tools (hidden Markov model) [1]. It belongs to the galanin family together with galanin (Gal), kisspeptin, and GALP proteins. This hormone is highly conserved among species (humans, rodents, fish, among others), indicating relevant roles in biological processes. Moreover, it is expressed in many tissues such as the liver, brain, adipose, stomach, and intestine [2,3], and has been related to different metabolic functions such as regulation of body weight, energy balance, gastrointestinal movements, glucose and lipid metabolism, and lipid storage [4,5,6,7]. Among galanin receptors, GALR2 and GALR3 have also been proposed for SPX action. While both Gal and SPX have an affinity for GALR2, mainly SPX is responsible for GALR3 activation [8,9]. These receptors are all G-protein-coupled receptors, but little is known about their expression. *Galr2* seems to be widely expressed in tissues, whereas *galr3* is more variable, with little or no expression [4,8,10].

In Type 2 diabetes mellitus (T2DM), for obese and insulin-resistant patients, SPX plasma levels are decreased [3,11,12,13]. Also, SPX levels correlate negatively with fasting blood glucose and lipid levels. In healthy women, SPX correlates negatively with age, body mass index (BMI), triglyceride (Tg) levels, and fasting glucose levels in plasma. Moreover, *spx* mRNA expression in adipose tissue (AT) from obese humans and mice is reduced [4,10]. On the other hand, in various obesity models, SPX treatment has been shown to improve metabolic parameters, adipocyte hypertrophy and inflammation, and macrophage infiltration in AT [4,6,10,14,15,16]. Also, in vitro studies in adipocytes and hepatocytes have shown that SPX favors lipolysis and inhibits glucose uptake and lipogenesis [4,17]. Regarding SPX receptors, Kim et al. and our group have found that *galr2* levels increase after an obesogenic diet administration, whereas different results have been found for *galr3*: contrary to Kim et al., who found increased *galr3* expression, we failed to detect *galr3* in epididymal AT [8,10]. In agreement with our results, Walewski et al. did not find *galr3* expression in human samples [4].

White AT (WAT) consists of different types of cells, including mature adipocytes, preadipocytes, fibroblasts, and endothelial and immune cells. In WAT, it is possible to distinguish two adipocyte phenotypes: those commonly known as white adipocytes, which store fat in a large unilocular lipid droplet, and beige/brite adipocytes, which resemble brown adipocytes, as they contain small multilocular lipid droplets. Beige adipocytes can dissipate energy into heat through the activity of the mitochondrial Uncoupling protein 1 (UCP1). Under cold and/or pharmacological conditions (β3-adrenergic receptor activation), white adipocytes can become beige adipocytes [18,19]. This process is known as browning, and its activation has widely been accepted as an effective strategy to treat both obesity and metabolic diseases. Browning promotes energy expenditure by thermogenesis activation, regulating body weight and glucose and lipid homeostasis [20,21].

Under basal conditions, beige adipocytes may remain in a latent/resting state, with low or almost no detectable UCP1 expression, similar to white adipocytes, but after a stimulus (cold or β-adrenergic receptor agonists), UCP1 expression is powerfully activated, and the beige phenotype becomes evident. In parallel, the differentiation of beige precursors may also be promoted [22,23]. During this differentiation, PRDM16, a transcriptional co-regulator, interacts with several DNA-binding transcriptional factors such as PPARγ and the C/EBPs family and induces PGC1α and COX8B expression, triggering mitochondrial biogenesis and mitochondrial electron transport activation, respectively. This results in increased UCP1 expression and activity [19,24].

Little is known about the role of SPX in thermogenesis. Recent studies have described decreased *ucp1*, *pgc1α*, and *prdm16* expressions in brown AT (BAT) and lower immunodetection of β3-adrenergic receptors in gonadal WAT from obese female mice treated with SPX for 50 days [25]. In the liver, results are contradictory. While one study has shown that SPX decreases *pgc1α* expression both in vivo and in vitro [26], another study has shown the opposite effect [27]. Thus, the aim of this study was to evaluate the role of SPX during WAT thermogenesis under both room temperature (RT) and cold-stimulated conditions in male mice.

## 2. Results

### 2.1. Metabolic Effect of SPX under Cold Conditions

**Figure 1:**
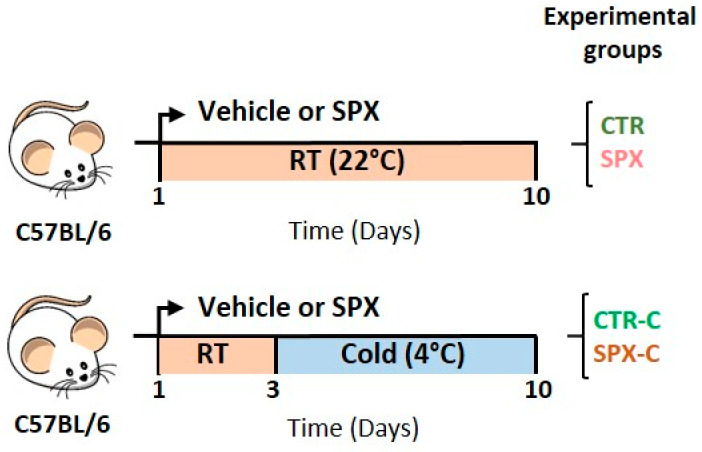
Experimental design and groups for the assessment of the role of SPX in WAT thermogenesis in vivo. Timelines show the experimental period in days. RT: room temperature.

**Figure 2:**
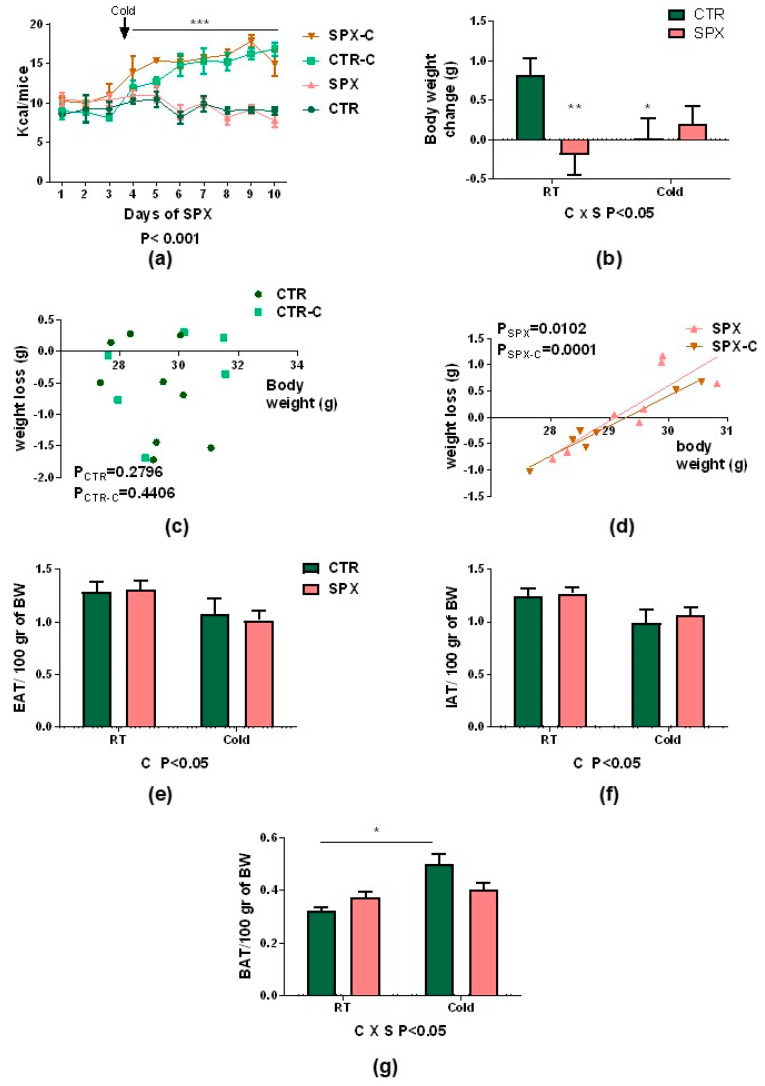
(**a**) Mean of daily caloric intake and (**b**) body weight change during the protocol. Pearson’s correlation between weight loss vs. initial body weight for (**c**) CTR and CTR-C and (**d**) SPX and SPX-C. A significant correlation was considered when *p* < 0.05. (**e**) EAT, (**f**) IAT, and (**g**) BAT mass percentages. For caloric intake, repeated measures ANOVA was performed. For other parameters evaluated, a two-way ANOVA was performed. The effects of SPX (S) and cold (C) and their interaction (C × S) were evaluated. If significant, their *p*-values are detailed at the bottom of each graph. When the interaction was statistically significant (*p* < 0.05), a group-to-group comparison was performed using the Bonferroni test. * *p* < 0.05, ** *p* < 0.01 vs. CTR, and *** *p* < 0.001 vs. CTR/SPX. Values are mean ± SEM (n = 6–9 mice per group).

Following the experimental protocol (Figure 1, caloric intake and body weight were recorded daily in all groups, both SPX-treated and controls under basal and cold conditions (SPX, CTR, SPX-C, and CTR-C, respectively). As expected, caloric intake increased when animals were exposed to cold, but no differences were observed due to the SPX treatment (Figure 2a, SPX-C and CTR-C vs. SPX and CTR; *p* < 0.001). On the other hand, at basal conditions (room temperature (RT)), SPX animals lost body weight vs. CTR ones, whereas after cold exposure, SPX-C mice lost less weight than their CTR-C counterparts (Figure 2b), evidenced by the variable interaction (C × S (cold × SPX), *p* < 0.05, two-way ANOVA). Then, we evaluated the correlation between the initial body weight and the body weight loss after SPX treatment. No correlations were found for CTR and CTR-C (Figure 2c). However, a significant positive correlation was observed in SPX mice under both basal and cold-stimulated conditions (Figure 2d, Pearson correlation, *p* < 0.01). This result indicates that the SPX treatment favors weight loss depending on the initial weight. Thus, animals with higher body weights were able to lose more weight than lean animals. When the AT depots were dissected and weighed (inguinal and epidydimal AT; IAT and EAT, respectively), the mass percentage determined after cold exposure was significantly lower (Figure 2e,f, C *p* < 0.05) for both pads. For BAT, a variable interaction was found (C × S *p* < 0.05), showing an increase in CTR-C vs. CTR, although SPX-C mice showed an intermediate value (Figure 2g).

**Figure 3:**
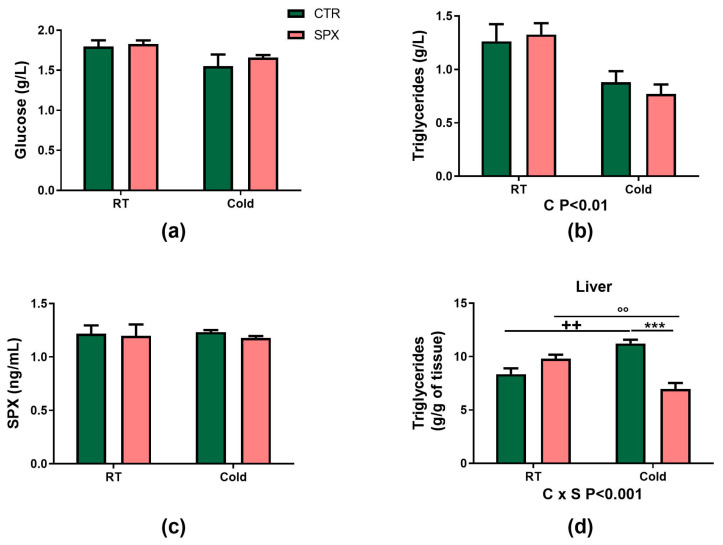
(**a**) Glucose, (**b**) triglycerides (Tg) and (**c**) SPX plasma levels, and (**d**) liver Tg content. The effects of cold (C) and SPX (S) and their interaction (C × S) were evaluated by a two-way ANOVA. If significant, their *p*-values are detailed at the bottom of each graph. When the interaction was statistically significant, group-to-group comparison was performed by the Bonferroni test. *** *p* < 0.001 vs. CTR-C, ++ *p* < 0.001 vs. CTR; °° *p* < 0.01 vs. SPX. Values are mean ± SEM (n = 6–7 mice per group).

Regarding the other metabolic parameters evaluated, the SPX treatment did not change the plasma glucose or Tg levels (Figure 3a,b). Nevertheless, under cold conditions, both groups significantly decreased the plasma Tg levels vs. basal conditions (C *p* < 0.01) (Figure 3a,b). Despite SPX administration, the plasma SPX levels did not differ between groups, as previously observed in other models from our laboratory (Figure 3c) [10]. As SPX is a short peptide and its half-life in plasma has not been previously assessed, we decided to evaluate the SPX levels in plasma at different times after intraperitoneal (ip.) injection in CTR mice in order to determine the highest level and the return to normal levels of circulating SPX. SPX’s half-life was short: 1 h after SPX injection, plasma SPX levels were two-fold higher than basal ones, and 3 h after SPX injection, the levels were already back to basal levels (Supplementary Figure S1).

The liver Tg content showed an interaction of variables (C × S *p* < 0.001). CTR-C mice had higher Tg levels than CTR (*p* < 0.01) and SPX-C (*p* < 0.001) mice (Figure 3d). Moreover, SPX-C mice had lower Tg levels than SPX mice.

### 2.2. SPX Decreased UCP1 Expression in AT of Treated Mice

**Figure 4:**
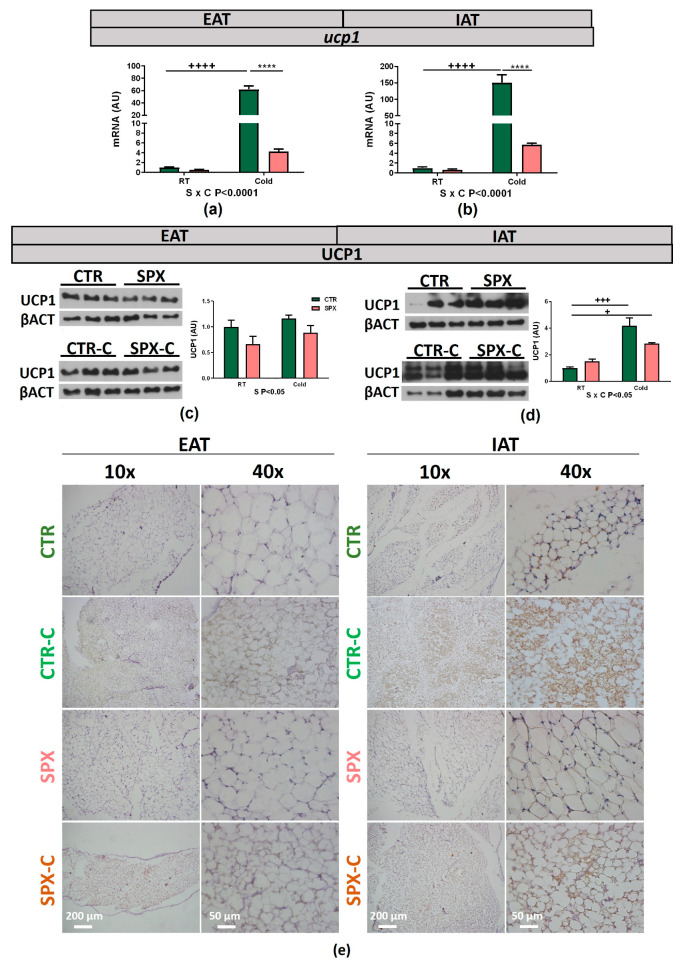
(**a**) EAT and (**b**) IAT *ucp1* mRNA expression. (**c**) EAT and (**d**) IAT UCP1 protein expression, representative figures, and quantitative analyses, respectively. Β-actin was used as the loading control. n = 4–6 samples per group were analyzed. In all cases, two-way ANOVA was performed for the factor (S or C) and the interaction (C × S), followed, if required, by the Bonferroni test. If significant, *p*-values are detailed below the graph. + *p* < 0.05, +++ *p* < 0.001, ++++ *p* < 0.0001 vs. CTR, **** *p* < 0.0001 vs. CTR-C. Data shown are mean ± SEM. (**e**) EAT and IAT UCP1 immunohistochemistry from CTR, SPX, CTR-C, and SPX-C mice. Images are representative of three experiments. Magnification: 10X and 40X. The 10X magnification shows the global UCP1+ signal (brown) and the distribution of multilocular adipocytes. Scale bars: 200 μm in 10X images; 50 μm in 40X images.

We further analyzed UCP1’s expression, a key factor involved in the thermogenic process. It is widely accepted that UCP1 increases upon cold stimulation. In our model, after 7 days of cold exposure, we found that *ucp1* mRNA expression was increased in all AT depots analyzed (IAT, EAT, and BAT). During SPX treatment, an interaction between variables (C × S *p* < 0.05) was found in both IAT and EAT. We found that *ucp1* expression in the CTR-C group was significantly increased vs. the CTR and SPX-C groups (*p* < 0.0001). This indicates that, under cold-stimulated conditions, SPX caused *ucp1* inhibition (Figure 4a,b). At RT, SPX inhibition was also significant, but only when a *t*-test was performed. On the other hand, the increase in *ucp1* levels in IAT was higher than in EAT from CTR-C mice, as expected, taking into account that, in mice, subcutaneous depots are thermogenically more active than visceral depots. In BAT, the cold variable was significant, being that *ucp1* expression increased under cold exposure despite the SPX treatment (supplementary Figure S2a; C *p* < 0.001). UCP1’s protein expression was then analyzed by Western blot and immunohistochemistry for EAT and IAT. Firstly, Western blot for EAT revealed that SPX reduced the UCP1 protein levels at both basal and cold conditions (Figure 4c, *p* < 0.05, SPX and SPX-C). In IAT, similar to that observed for the UCP1 mRNA levels, an interaction was noticed (C × S *p* < 0.05), indicating an inhibitory SPX effect under stimulated thermogenic conditions. The post-test analysis showed that UCP1 expression in CTR-C was increased vs. CTR (*p* < 0.001) and that UCP1 expression in SPX-C mice showed an intermediate value between that in the CTR and CTR-C mice, thus indicating a partial decrease in UCP1 levels (Figure 4d). As expected, the immunohistochemistry microphotographs showed that UCP1 staining of IAT was more evident than for EAT in both the CTR and SPX groups (Figure 4e). However, in the SPX groups, UCP1 expression was lower than in the CTR groups.

### 2.3. SPX Modulates Mitochondrial DNA and Gene Expression under Cold Conditions

**Figure 5:**
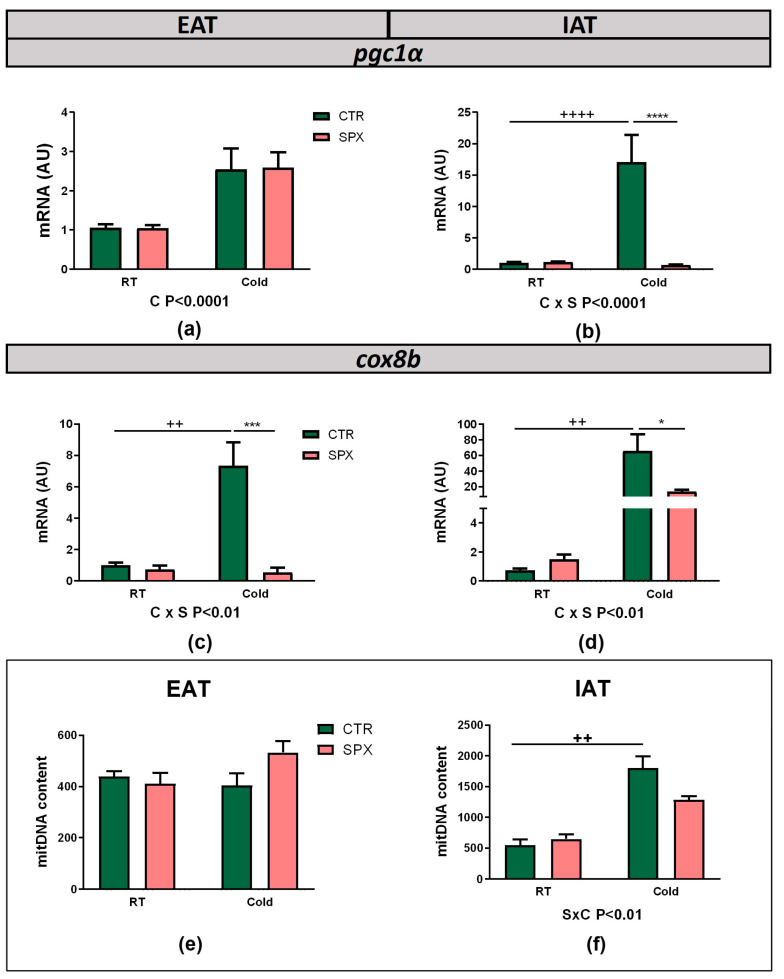
*Pgc1α* (**a**,**b**) and *cox8b* (**c**,**d**) mRNA expression in EAT and IAT depots. Mitochondrial DNA content quantification in (**e**) EAT and (**f**) IAT. Data shown are mean ± SEM. In all cases, two-way ANOVA was performed for factor (S or C) and interaction (C × S) analysis and, if significant, the *p*-values are detailed below the graph. When the interaction was statistically significant, a group-to-group comparison was performed by the Bonferroni test. ++ *p* < 0.01, ++++ *p* < 0.001 vs. CTR; * *p* < 0.05, *** *p* < 0.001, **** *p* < 0.0001 vs. CTR-C (n = 4–6 mice per group).

WAT browning activation is generally accompanied by an increase in the number of mitochondria and mitochondrial mRNA and protein levels. For this reason, we evaluated the mRNA expression of *pgc1α* and *cox8b*, the main genes involved in mitochondrial biogenesis and the electron transport chain, together with the mitochondrial DNA content (mitDNA). Under cold conditions, *pgc1α* levels increased in EAT for both the control and treated groups (Figure 5a, C *p* < 0.0001). In IAT, the interaction of variables was found (C × S *p* < 0.001). Further, pgc1α expression was increased in the CTR-C vs. CTR mice and inhibited due to SPX in cold conditions (SPX-C vs. CTR-C, *p* < 0.001) (Figure 5b). In BAT, no changes were observed in the *pgc1α* expression (Supplementary Figure S2b). The *Cox8b* levels revealed a significant interaction between variables in EAT, IAT, and BAT (Figure 5c,d; Supplementary Figure S2c, C × S *p* < 0.001). In all tissues, the post-test showed a significant increase in the CTR-C vs. CTR and SPX-C mice. Thus, these results again showed an inhibitory effect of SPX under cold conditions (Figure 5c,d and Figure S2b,c).

The mitDNA content showed no changes in EAT, but the interaction between variables was significant in IAT (Figure 5e,f, C × S *p* < 0.01). The post-test showed that the mitochondrial content in CTR-C mice was increased vs. CTR ones, wherein that in SPX-C mice remained at an intermediate level between that observed in CTR and CTR-C, indicating a partial inhibition, although no significant differences were found (Figure 5f).

### 2.4. SPX Treatment Did Not Modulate the Percentage of Beige Precursor Adipocyte Cells

**Figure 6:**
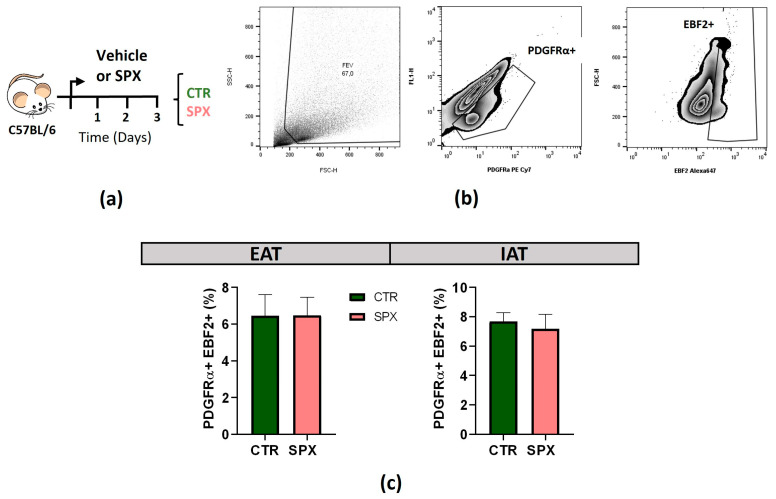
(**a**) Experimental design for beige precursor quantification prior to cold exposure. (**b**) Gating strategy for the identification of beige precursors (PDGFRα+; EBF2+) in the SVF of EAT and IAT from CTR and SPX mice after 3 days of SPX treatment (S). The cells studied were selected, eliminating the immune cells, dead cells, and debris according to size/volume (SSC-H) and complexity (FSC-H), resulting in a reduced SVF. We first identified PDGFRα+ cells (PDGFRα+ PE/Cy7). Then, within PDGFRα+, we selected EBF2+ cells (Ebf2 AlexaF 647). (**c**) Quantification of the abundance (%) of beige precursors (PDGFRα+; EBF2+) in the SVF of EAT and IAT from CTR and SPX mice. n = 4, mean ± SEM. The effects were evaluated by *t*-test. The value was statistically significant when *p* < 0.05.

In order to determine whether SPX could also inhibit the number of beige adipocyte precursors, we isolated the stromal vascular fraction (SVF) from EAT and IAT of mice treated or not with SPX for 3 days prior to cold exposure (CTR and SPX; Figure 6a). Cells were assessed by flow cytometry for beige precursors using the following markers: PDGRFα+ and EBF2+ (Figure 6b [28]). Of notice, no changes were observed in either of the two AT depots studied (Figure 6c).

### 2.5. Modulation of the Expression of spx and galr2/3 Receptors upon Cold Exposure

**Figure 7:**
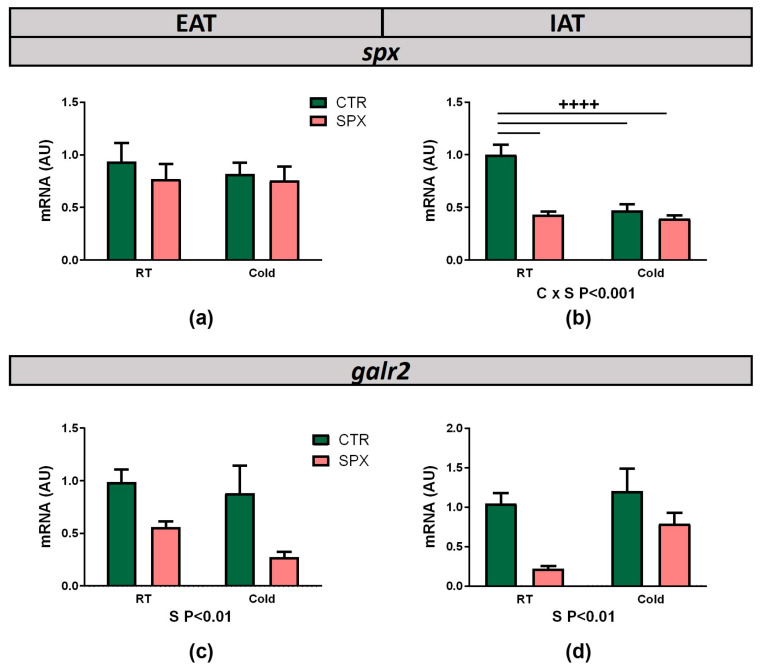
mRNA expression of *spx* (**a**,**b**) and *galr2* (**c**,**d**) in EAT and IAT depots, respectively. Data shown are mean ± SEM. In all cases, two-way ANOVA was performed for factor (C or S) and interaction (C × S) analysis and, if significant, the *p*-values are detailed below the graph. When the interaction was statistically significant, a group-to-group comparison was performed by the Bonferroni test. ++++ *p* < 0.001 vs. CTR (n = 4–6 mice per group).

Since no information was found about the expression of *spx* and its receptors (*galr2/3*) upon cold exposure, our first approach was to evaluate the gene expression in WAT and BAT depots. No changes in *spx* mRNA expression were observed in EAT and BAT, whereas a significant interaction between variables was noticed in IAT (Figure 7a,b, C × S *p* < 0.001; Supplementary Figure S3a). The post-test showed that, under cold conditions, SPX mice showed a lower *spx* expression than CTR mice (Figure 7b). Regarding *galr2* mRNA expression, we found that, in both WAT depots, the effect of SPX was statistically significant, being that *galr2* mRNA expression decreased in both groups treated with SPX (Figure 7c,d, S *p* < 0.01). However, no differences were found in BAT (Supplementary Figure S3b). *Galr3* mRNA expression was barely detectable. In EAT and BAT, no *galr3* expression was detected, whereas in IAT, *galr3* expression was variable and very low in RT samples (CTR and SPX). In cold-exposed groups (CTR-C and SPX-C), *galr3* was almost not detectable.

## 3. Discussion

This study showed, for the first time, that SPX is able to inhibit the thermogenic process in male mice exposed to cold conditions. As expected, when animals were exposed to cold, the browning process was activated, and different markers, such as *pgc1α*, *cox8b*, and *ucp1*, were increased, but SPX treatment reduced their levels mainly in IAT. Moreover, the UCP1 protein quantification showed an intermediate value between CTR and CTR-C in IAT after SPX treatment and cold exposure. Additionally, mitochondrial DNA was reduced together with mitochondrial gene expression markers. Finally, no alterations were observed in the number of beige precursors after three days of SPX treatment.

Currently, there is little information about the role of SPX during thermogenesis in AT. In line with our work, Sherman et al. showed that obese female mice treated with SPX for 50 days had a reduced AT thermogenic process [25]. Briefly, these authors found that the UCP1 expression was downregulated in BAT and that the β3-adrenergic receptor was downregulated in gonadal WAT. Since nest building is an innate behavior that demands energy for thermoregulation [29,30], they evaluated the nest-building complexity of mice and found that SPX mice built more complex nests (scoring system according to Deacon et al. [31]) at room temperature. This behavior was normalized at thermoneutral temperatures, indicating some compensatory mechanism for their reduced ability for thermoregulation. In our work, we observed a marked decrease in the thermogenic markers, mainly upon cold stimulation in WAT, and did not observe differences in BAT under basal or cold conditions. These differences may be due to the mouse model chosen (obese vs. control mice), the extension of the treatment (50 days vs. 10 days), and/or sexual dimorphism (female vs. male). Furthermore, it is important to highlight that this study only measured the β3-adrenergic receptor in WAT at RT, but not after cold exposure, where thermogenesis is highly active. On the other hand, two studies in the liver of obese mice have shown that SPX treatment improves many metabolic parameters (Tg, glucose, and glucose tolerance test) and liver steatosis but showed different results regarding PGC1α expression. In one of them, PGC1α was increased [27], whereas in the other, the results were opposite [26]. These discrepancies may be due to the extension of the SPX treatment (8 weeks vs. 3 weeks) or to the animal model studied (rats vs. mice) [26,27]. Moreover, Gu et al. related the decrease in PGC1α and other proteins to a reduction in the gluconeogenic pathway, which favors metabolic improvement in obesity. In contrast, Wang et al. related PGC1α induction to mitochondrial respiration and fatty acid oxidation. Thus, further studies should be performed. Regarding our present work, we observed a reduction in *pgc1α* after SPX treatment in IAT from cold-exposed animals, like Gu et al., in the liver [26].

Beige adipocytes may be generated through two different mechanisms: adipogenesis, de novo, from precursor stem cells, and the transdifferentiation process, which involves the conversion/reprogramming of white adipocytes. Regarding the former, different studies have shown the presence of different adipocyte precursors that have different thermogenic capacities when differentiated in vitro [22]. Other studies have observed that PDGFRα+ cells are bipotential precursors, being able to differentiate into either white or beige adipocytes depending on the stimulus. Then, only PDGFRα+ and EBF2+ cells were characterized as precursors with beige differentiation potential [28,32,33]. For the transdifferentiation process, beige adipocytes originate from differentiated white adipocytes under a certain stimulus (e.g., β3-adrenergic stimulation), where an intermediate adipocyte phenotype is observed (paucilocular adipocytes) [32,34,35] and a reprogramming process is involved (mithophagy/autophagy) [36]. Therefore, we evaluated whether SPX modulates the number of beige precursors of the SVF prior to cold exposure but found no effect. These results indicate that SPX may not be involved in the modulation of beige precursors. On the other hand, it is known that SPX does not affect the viability or proliferation of preadipocytes, although it inhibits the terminal differentiation of white 3T3-L1 adipocytes in vitro [17]. It is known that the ability to generate beige adipocytes by adipogenesis depends on both the number and adipogenic potential of precursors (competency) [37,38]. In the present study, we evaluated only the effect of SPX on the number of precursors; therefore, we cannot exclude that SPX modulates competency. Further studies should be performed to clarify this issue.

It is known that, in mice, subcutaneous WAT depots have a higher thermogenic capacity due to enhanced UCP1 expression levels compared to visceral WAT depots [22,39,40]. In the present study, we observed that, at RT, IAT had a higher UCP1 expression than EAT and that the extent of increase upon cold exposure was higher in IAT, but also that SPX treatment was able to reduce the UCP1 expression in both depots at basal and cold-stimulated conditions. No changes were observed for BAT. Moreover, *pgc1α* was negatively modulated by SPX only in IAT, while the *cox8b* levels were reduced in all AT depots in SPX-C mice. Thus, these results indicate that SPX may inhibit thermogenesis in WAT depots, being AT-dependent, producing a more marked effect in IAT.

Regarding the SPX receptors, little is known, especially after SPX treatment. It is known that GALR2 increases during obesity, but different results have been found for GALR3. While some authors have described an increase in GALR3 expression, others, like us and Waleski et al., have found very low or no expression [4,8,10]. Here, we observed that upon SPX treatment, *galr2* decreased regardless of the cold exposure, and *galr3* was barely detectable. Further studies with a GALR2 antagonist should be performed to better understand the mechanism involved in this phenomenon and, importantly, the signaling pathways activated (PI3K/AKT/Fox01 or MAPK) [41,42]. To our knowledge, there are no studies about how the SPX-GalR complex is affected during cold exposure. We found, at least in our experimental design, that plasma SPX was not changed by cold but that *spx* expression in IAT was decreased. We also found that *galr2* in both WAT depots was decreased only after SPX treatment. In this study, we showed, for the first time, the SPX kinetics after the treatment of mice with SPX, whose half-life is short but whose effect persists in time (Supplementary Figure S1). Similar results have been shown in vitro by Reyes Alcaraz et al. [9].

In obese patients, plasma SPX levels are decreased, and different correlations between these levels and different metabolic parameters have been found. In humans, SPX plasma levels negatively correlate with Tg levels, BMI, and HOMA-IR [11,13,43]. Moreover, others and ourselves have previously shown that SPX treatment favors weight loss in obese mice [4,10,15,26], with the exception of one work in obese female mice [25]. In this study, we observed the same behavior previously reported by us, with SPX as a weight loss regulator [10]. We found again a significant correlation between the initial weight (before SPX treatment) and weight loss after the SPX treatment in both groups (SPX and SPX-C), regardless of cold exposure.

Different obesity models in male mice have shown improvements in many metabolic parameters and inflammation of AT and the liver after SPX treatment [4,6,10,26,27,44]. Briefly, SPX decreased AT hypertrophy and liver steatosis, favored by in vitro lipolysis, improved insulin sensitivity, and reduced inflammatory macrophage infiltration and inflammatory gene markers in AT [4,6,7,10,26,27]. On the other hand, activating thermogenesis has been proposed as a treatment for obesity since it favors energy expenditure and improves many detrimental effects of obesity [20,21,45]. Moreover, considering that the abdominal depot is less thermogenic than the subcutaneous one and that it is widely accepted that visceral AT is metabolically more active than the subcutaneous one and modulates many adipokines in plasma [46,47], we may hypothesize that the beneficial metabolic effect of SPX during obesity, principally in visceral AT, primes to the negative thermogenic regulation in this depot. Thus, further studies should be performed to evaluate the relationship between SPX, obesity, and thermogenesis and the mechanisms involved in subcutaneous and visceral WAT. Likewise, other thermogenic Ucp1-independent processes need to be evaluated, as they may compensate for the Ucp1 thermogenic mechanism [48,49].

In conclusion, in this study, we evaluated the global effect of SPX on browning under basal and cold-stimulated conditions, including the overall response of the different tissues targeted by SPX. However, using an in vivo approach, we were not able to evaluate the direct effect of SPX on mature adipocytes and/or beige precursors or the effect of antagonist receptors, e.g., using the in vitro models. Despite this, our work is the first approach to evaluating the role of SPX in thermogenesis. Briefly, we found that mice exposed to cold showed less activation of the browning process when treated with SPX, especially in IAT, which is considered the most thermogenically active WAT. Additionally, IAT responded to the SPX treatment to a higher extent than EAT, decreasing the thermogenic gene program. Finally, SPX inhibited the UCP1-dependent thermogenic process in WAT, mainly upon cold exposure. We believe that these novel findings open the door for further investigations of the role of SPX during the activation and maintenance of the thermogenic process.

## 4. Materials and Methods

### 4.1. Animals and Treatment

Normal adult male C57BL/6 mice (four months of age) were kept in a temperature-controlled environment (20–22 °C and a fixed 12 h light/12 h dark cycle; lights on at 07:00 h) and fed ad libitum with Purina commercial rat/mouse chow (Ganave, Buenos Aires, Argentina, L0723/1). Mice were injected with SPX or PBS (29 µg/kg/day, Tocris, Bristol, UK) for ten days (the SPX and CTR groups, respectively). On the third day of the protocol, each group was subdivided into two other groups: one exposed to cold (4 °C, CTR-C and SPX-C, n = 12 mice for each group) and another one housed at RT (CTR and SPX, n = 15 mice for each group) (Figure 1). Food intake and body weight were measured every day. On the experimental day (day 10), the mice were euthanized under non-fasting conditions (08:00 to 09:00 h), and their trunk blood was collected; plasma samples were then frozen (–80 °C) until the metabolite measurement. Inguinal AT (IAT, subcutaneous depot), epididymal AT (EAT, visceral depot), and brown AT (BAT, subcutaneous depot) were aseptically dissected and weighed. These depots were kept at –80 °C for further procedures. Animals were euthanized according to protocols for animal use, in agreement with the NIH guidelines for the care and use of experimental animals. All experiments were approved by our Institutional Animal Care Committee (Approval code: 6NE-2023).

### 4.2. Peripheral Metabolite Measurements

Circulating glucose (Glu) and triglyceride (Tg) levels were measured using commercial kits (Wiener Lab., Rosario, Argentina). Plasma SPX was measured using a commercial ELISA kit (SPX: Phoenix pharmaceutical EK-023-81).

### 4.3. Liver Lipid Content

Liver tissue (50 mg) was homogenized in a 5% solution of 500 µL Triton X-100 in PBS. The homogenate was incubated at 80–100 °C for 5 min and centrifuged at 10,000× *g* for 10 min. The Tg levels were measured in the supernatants using a commercial kit (Wiener Lab., Rosario, Argentina).

### 4.4. RNA Isolation and Quantitative Real-Time PCR (qRT-PCR)

Total RNA was isolated from cells by the Trizol extraction method (Invitrogen, Life Technology, Waltham, MA, USA) and reverse-transcribed using random primers (250 ng) and RevertAid Reverse Transcriptase (200 U/µL, Thermo Scientific, Waltham, MA, USA). The cDNA (2 μL) was amplified with HOT FIRE Pol EvaGreenqPCR Mix Plus (Solis BioDyne, Tartu, Estonia) containing 0.5 μM of each specific primer, using a Rotor-Gene Q (Qiagen, Hilden, Germany). PCR efficiency was near 1. The expression levels were analyzed for β-actin (*βactin*, reporter gene), Uncoupling Protein 1 (*ucp1*), proliferator-activated receptor gamma coactivator-1 alpha (*pgc1α*), cytochrome-c oxidase subunit 8b (*cox8b*), galanin receptors 2 and 3 (*galr2* and *galr3*, respectively), and Spexin (*spx*). The designed primers are shown in alphabetical order in Table 1. The relative changes in the expression level of each gene (ΔΔCt) were calculated by the ΔCt method. 

### 4.5. Western Blot

For protein analysis by Western blot, IAT and EAT were lysed in a RIPA buffer supplemented with a protease and phosphatase inhibitor cocktail. Protein contents were quantified with the bicinchoninic acid method (Thermo Scientific, Pierce™ BCA Protein Assay Kit). Total proteins (20 μg) were separated by 12% sodium dodecyl sulphate-polyacrylamide gel (SDS-PAGE) and transferred to polyvinylidene fluoride membranes (PVDF). Membranes were blocked with 1–5% BSA in TBS-T and incubated overnight with anti-βactin (Cat# bs-0061R, lot# AH01032107; Bioss antibodies, Woburn, MA, USA, 1/4000) and anti-UCP1 (Cat# ab-10983, lot# GR214286-5; Abcam, Cambridge, UK, 1/3500). Peroxidase-conjugated anti-rabbit antibody (Cat# sc-2004, lot# 22905; Santa Cruz, Dallas, TX, USA, 1/10,000) was used as the secondary antibody. The bands were visualized using a chemiluminescence reagent and autoradiographed (Carestream Medical X-ray Green/MXG film). Finally, relative protein levels were calculated using Image J Fiji software 1.54f.

### 4.6. Mitochondrial DNA Quantification

Tissue from the EAT and IAT depots were homogenized with lysis buffer (40 mM EDTA, 50 mM NaCl, 100 mM Tris, 0.2% SDS) in PBS (2:1) and, for DNA fragmentation, samples were sonicated (38 kHz ± 10% for 5–10 s). Proteins were precipitated with 5.4 M NaCl and centrifuged at 8000 rpm for 20 min, and soluble DNA was precipitated with cold isopropyl alcohol. Finally, the DNA was dissolved in TE buffer and quantified by Nanodrop at 260 nm. For qPCR reactions, as detailed above, 5 ng of DNA was used for the rRNA-16S (mitochondrial gene) and β2-microglobulin (nuclear gene) determinations (primer sequences are detailed in Table 1). The relative changes in the DNA mitochondrial content were calculated by 2[2, −ΔΔCt] [50,51].

### 4.7. Immunohistochemistry

For the EAT and IAT histological studies, freshly dissected EAT pads were fixed in 4% paraformaldehyde, then washed with tap water, immersed in a series of graded ethanol (70, 96, and 100%), and clarified in xylene before paraffin embedding. Four-micrometer sections were taken from different levels of the blocks in slides coated with gelatin (0.3% gelatin, 0.05% Chromium Potassium Sulfate). Afterward, sections were deparaffinized, rehydrated, and subjected to heat-induced antigen retrieval (sodium citrate buffer, pH = 6). The endogenous peroxidase activity was blocked with 0.3% H_2_O_2_, and unspecific binding of the primary antibody was blocked with 10% fetal bovine serum (FBS) and 1% BSA in TBS. The samples were then incubated with the primary antibody (Ucp1, 1:500, Abcam) at 4 °C overnight in a humidified chamber. The primary antibody was washed and incubated with HRP-conjugated goat anti-rabbit secondary antibody (1:500, Santa Cruz) for 1.5 h. Then, the samples were developed with 0.05% DAB (Sigma, San Luis, MO, USA) and 0.015% H_2_O_2_ in PBS for 10 min and counterstained with hematoxylin for nuclei visualization. Finally, the samples were dehydrated and mounted with Canada balsam. Controls of unspecific binding of the primary antibody and activity of the endogenous peroxidase were made for each group and replica. Bright-field images were acquired with 10×/0.30 and 40×/0.65 objectives using a Nikon Eclipse 50i and a DS-Ri1 Nikon digital camera with a 0.55× adapter in the same optical and light conditions.

### 4.8. EAT and IAT Stromal Vascular Fraction Cell Isolation

Fresh EAT pads were dissected, weighed, and digested with collagenase, as previously reported [52]. Briefly, fat tissue was minced and digested using 1 mg/mL of the collagenase solution in DMEM (at 37 °C for 40–60 min). After centrifugation (1000 rpm for 15 min), the SVF pellets were filtered (in a 50-µm-mesh nylon cloth) and washed with DMEM (x3). SVF cells were then resuspended in DMEM supplemented with 10% (*v*/*v*) FBS, HEPES (20 nM), 100 IU/mL of penicillin, and 100 µg/mL of streptomycin (basal medium) and reserved for the flow cytometry protocol.

### 4.9. Flow Cytometry Analysis

The SVF was prepared as reported above and counted and filtered through a 70-μM cell strainer. To block unspecific binding, the cells were resuspended in 10% inactivated FBS in FACS buffer (1% BSA in PBS) for 10 min. Then, the cells were incubated with the surface antibody anti-Pdgfrα (CD140a)-PE/Cy7 (1:200, BioLegend, San Diego, CA, USA) and washed twice with FACS buffer, followed by centrifugation (300× *g*, 5 min). Fixation was performed with 2% paraformaldehyde in PBS for 20 min at room temperature. After washing, the cells were subjected to permeabilization with 0.1 % Triton X100 in PBS for 15 min at room temperature. Finally, the cells were incubated with the intracellular antibody anti-Ebf2-Alexa Fluor 647 (1:400, Bioss antibodies, Woburn, MA, USA) and washed once. Antibody incubations were performed on ice for 30 min. The samples were analyzed on a FACS Calibur flow cytometer (BD bioscience, Franklin Lakes, NJ, USA). Analysis was performed using FlowJo software 10.8.1.

## 5. Statistical Analysis

Before applying statistical analysis, data normality was assessed with a Shapiro–Wilk test. The normally distributed data were represented as the mean ± standard error of the mean (SEM). Two-way ANOVA was performed for factorial analysis, taking the effects of SPX and cold as the variables. When the interaction between the two factors (cold × SPX, C × S) was statistically significant (*p* < 0.05), a post hoc test was performed for comparisons between the groups (Bonferroni’s test) [53]. Pearson’s correlation was performed for Figure 2c,d. Further, *p* < 0.05 was considered significant. To determine the statistical differences in the experiments with one independent variable and two groups, the paired Student’s *t*-test was applied for normally distributed data (Figure 6c). The specific statistical tests applied and sample sizes are indicated in the corresponding figure legend. All statistical tests were performed and graphed using GraphPad Prism 8.0 (GraphPad Software Inc., San Diego, CA, USA).

## Figures and Tables

**Table 1:** Primers used for qPCR.

Gene	Sequence (5′-3′)	GBAN	Product Size (bp)
*βactin*	Fw: TTGCAGCTCCTTCGTTGCC	NM_007393.5	189
	Rv: ACCCATTCCCACCATCACAC		
*ucp1*	Fw: GGATTGGCCTCTACGACTCAG	NM_009463.3	150
	Rv: ACCCATTCCCACCATCACAC		
*pgc1α*	Fw: AAAAGCTTGACTGGCGTCAT	NM_008904.3	199
	Rv: ACACCACTTCAATCCACCCAG		
*cox8b*	Fw: CCGAGAATCATGCCAAGGCT	NM_007751.3	174
	Rv: TCCTGCTGGAACCATGAAGC		
*galr2*	Fw: CTGCAAGGCCGTTCATTTCC	NM_010254.4	86
	Rv: CCAGATACCTGTCCAGCGAG		
*galr3*	Fw: GGCCGTCTCAGTGGATAGGT	NM.015738.2	137
	Rv: AGCTTAGGTAGGGCGCGGA		
*spx*	Fw: TCCTTCTCCTGGTGCTGTCT	NM_001242345.1	187
	Rv: TCTGGGTTTCGTCTTTCTGG		
*β2-microglobulin*	Fw: GTGCCTCTTTCCCCTCTCTT	NC_000068.7	96
	Rv: TCCACCCTGTAGCCTCAAAG		
*rRNA-16S*	Fw: AAGTTTAACGGCCGCGGTAT	NC_005089.1	97
	Rv: AGTTGGACCCTCGTTTAGCC		

Fw: forward; Rv: reverse; GBAN: GenBank Accession Number; amplicon length in bp.

## Data Availability

Data is contained within the article and supplementary material.

## Supplementary Materials

The following supporting information can be downloaded at: https://www.mdpi.com/article/10.3390/ijms25031767/s1.
