# Supplementary material for: Role of Spexin in White Adipose Tissue Thermogenesis under Basal and Cold-Stimulated Conditions
Source: Int J Mol Sci. 2024 Feb 1;25(3):1767. doi: 10.3390/ijms25031767 (PMC10855774; doi:10.3390/ijms25031767)
Supplement: Supplementary file 1 [file ijms-25-01767-s001.zip › ijms-2810266-supplementary.pdf]

Supplementary Figures

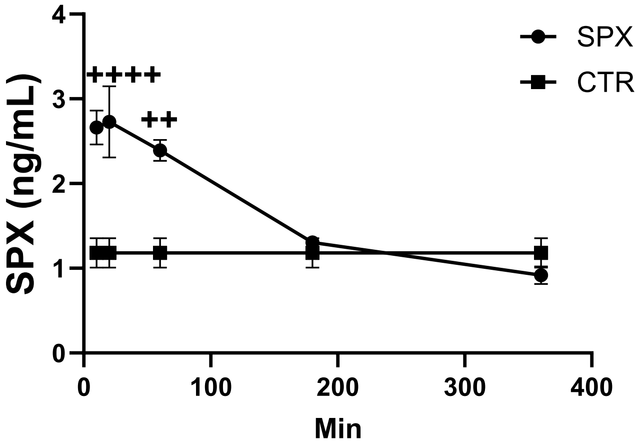

Supplementary figure 1. SPX levels (ng/ml, EIA Phoenix pharmaceutical) after intraperitoneal injection with SPX (29µg/kg). or Vehicle in mice Different time points were evaluated after ip. Injections (10, 20, 60, 180, 360 minutes). ++++P<0.0001, ++P<0.01 vs. CTR.

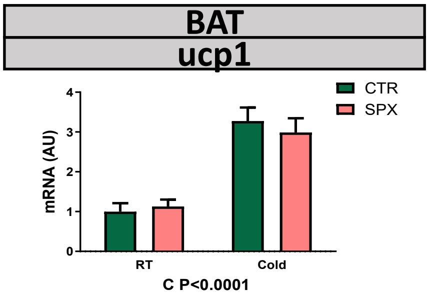

(a)

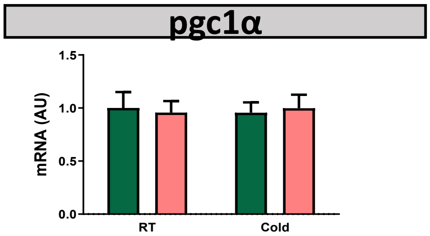

(b)

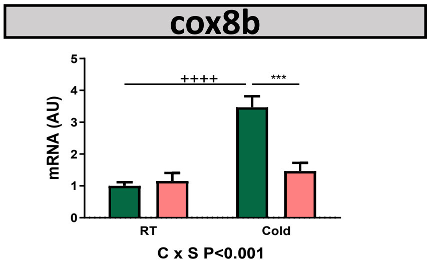

(c)

Supplementary Figure 2. ucp1 (**a**), pgc1*α* (**b**) and cox8b (**c**) mRNA expression in BAT. In all cases, two-way ANOVA was performed for factor (S or C) and interaction (S x C) analysis and detailed below the graph only when significant.. When interaction is statistically significant, group to group comparison was performed by Bonferroni test. ++++ P<0.0001 vs. CTR and ***P<0.001 vs. CTR-C (n=4-6 mice per group). Data was showed as mean ± SEM.

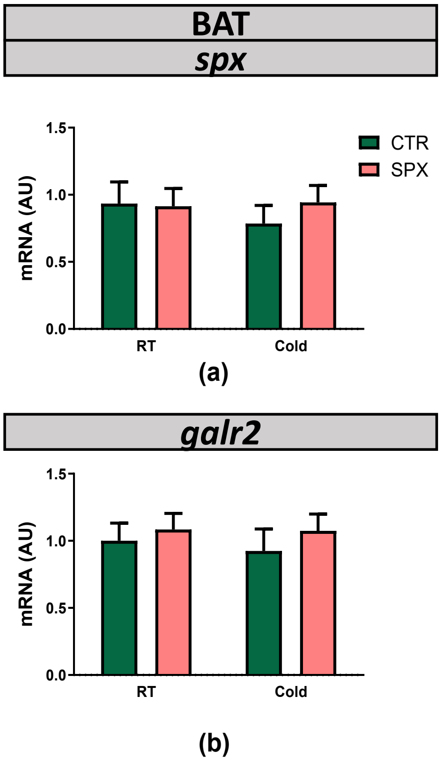

Supplementary Figure 3. (**a**) *galr2* and (**b**) *spx* mRNA expression in BAT. In all cases, two-way ANOVA was performed for factor (S or C) and interaction (S x C) analysis and detailed below the graph only when significant. When interaction is statistically significant, group to group comparison was performed by Bonferroni test (n=4-6 mice per group). Data was showed as mean ± SEM.
